# Age-Related Decline of Precision and Binding in Visual Working Memory

**DOI:** 10.1037/a0033236

**Published:** 2013-08-26

**Authors:** Muy-Cheng Peich, Masud Husain, Paul M. Bays

**Affiliations:** 1 Sobell Department of Motor Neuroscience and Movement Disorders, UCL Institute of Neurology and Institute of Cognitive Neuroscience, Queen Square, London, United Kingdom and University of Paris, Créteil, France; 2 UCL Institute of Neurology and Institute of Cognitive Neuroscience, Queen Square, London and Nuffield Department of Clinical Neurosciences and Experimental Psychology, University of Oxford, United Kingdom; 3 UCL Institute of Neurology and Institute of Cognitive Neuroscience, Queen Square, London

**Keywords:** aging, visual short-term memory (STM), resources, precision, binding

## Abstract

Working memory declines with normal aging, but the nature of this impairment is debated. Studies based on detecting changes to arrays of visual objects have identified two possible components to age-related decline: a reduction in the number of items that can be stored, or a deficit in maintaining the associations (*bindings*) between individual object features. However, some investigations have reported intact binding with aging, and specific deficits arising only in Alzheimer’s disease. Here, using a recently developed continuous measure of recall fidelity, we tested the precision with which adults of different ages could reproduce from memory the orientation and color of a probed array item. The results reveal a further component of cognitive decline: an age-related decrease in the resolution with which visual information can be maintained in working memory. This increase in recall variability with age was strongest under conditions of greater memory load. Moreover, analysis of the distribution of errors revealed that older participants were more likely to incorrectly report one of the unprobed items in memory, consistent with an age-related increase in misbinding. These results indicate a systematic decline with age in working memory resources that can be recruited to store visual information. The paradigm presented here provides a sensitive index of both memory resolution and feature binding, with the potential for assessing their modulation by interventions. The findings have implications for understanding the mechanisms underpinning working memory deficits in both health and disease.

A deterioration of short-term memory (STM) is a well-established feature of normal aging in humans (Craik, Luo, & Sakuta, 2010; D’Esposito & Gazzaley, 2011; Iachini, Iavarone, Senese, Ruotolo, & Ruggiero, 2009; Light, 1991; Reuter-Lorenz & Sylvester, 2005; Salthouse & Babcock, 1991). Considering the global aging of the world’s population, quantifying the decline of cognitive functions such as visual working memory (VWM) and understanding the mechanisms underlying it is of growing importance. Previous studies of age-related decline in VWM have sought to identify specific components of recall of visual information that deteriorate with age. These studies have predominantly been based on change detection tasks, in which observers are presented in sequence with two versions of an array of visual elements (e.g., colored squares) and must decide whether they are the same or different.

Change detection results in younger (university-age) participants have been interpreted as indicating a fixed memory capacity that can maintain a small number of integrated object representations (Luck & Vogel, 1997; Pashler, 1988; Vogel, Woodman, & Luck, 2001). In older participants, this approach has identified two putative components of the age-related decline in visual memory: a decrease in the number of whole objects that can be held in memory, and a failure to maintain the associations that bind individual visual features into objects (Bopp & Verhaeghen, 2009; Brockmole, Parra, Sala, & Logie, 2008; Cowan, Naveh-Benjamin, Kilb, & Saults, 2006; Mitchell, Johnson, Raye, & D’Esposito, 2000; Olson et al., 2004; Parra, Abrahams, Logie, & Sala, 2009; Sander, Werkle-Bergner, & Lindenberger, 2011a).

However, other investigators have found no decline in feature binding in healthy older people (Brockmole et al., 2008; Parra, Abrahams, Logie, et al., 2009) but have instead observed a specific failure of binding in Alzheimer’s disease (Parra, Abrahams, Fabi, et al., 2009; Parra, Abrahams, Logie, & Della Sala, 2010; Parra, Abrahams, Logie, Méndez, et al., 2010). Indeed, it has been argued that feature misbinding in working memory might be the earliest cognitive marker of disease onset (Parra, Abrahams, Logie, & Della Sala, 2010; Parra, Abrahams, Logie, Méndez, et al., 2010; Parra, Abrahams, Fabi, et al., 2009). The implications of such a proposal in terms of screening for early Alzheimer’s disease and distinguishing such cases from normal healthy people are obviously profound. It would, therefore, be important to establish whether maintenance of feature binding in working memory is truly invariant with age.

In contrast to the binary (correct or incorrect) responses obtained in tasks such as change detection, methods for examining the *precision* with which visual information is stored are an increasingly prominent feature of working memory research in younger adults (Bays, Catalao, & Husain, 2009; Bays, Gorgoraptis, Wee, Marshall, & Husain, 2011; Bays & Husain, 2008; Gorgoraptis, Catalao, Bays, & Husain, 2011; Huang, 2010; Lakha & Wright, 2004; Palmer, 1990; Wilken & Ma, 2004; Zhang & Luck, 2008; Zokaei, Gorgoraptis, Bahrami, Bays, & Husain, 2011). More recently, this approach has profitably been extended to children (Burnett et al., 2012) and nonhuman primates such as macaque monkeys (Elmore et al., 2011; Lara & Wallis, 2012). Here we apply the approach to older human participants, using a recently introduced dual-feature working memory protocol (Bays, Wu, & Husain, 2011). This task allows us to quantify both the resolution with which participants recall pairs of visual features and the fidelity with which they maintain feature bindings.

Our results indicate that the precision with which visual features can be maintained in memory declines with aging. When multiple items are held in memory, older individuals also demonstrate a significantly increased frequency of misreporting errors, in which they incorrectly report a visual feature belonging to one of the uncued items in the array. These errors are consistent with an age-related impairment in maintaining feature bindings. Our findings have implications for the understanding of normal aging as well as for the use of binding as a marker for Alzheimer’s disease.

## Method

### Experimental Procedures

A total of 60 participants (25 male, 35 female) with ages in the range 19 to 77 years took part in the study after giving informed consent. Participants were recruited by e-mail invitation to individuals who had signed up to Psychology and Cognitive Neuroscience Department subject pools. Invitations were targeted to recruit an even distribution of ages in the subject group. Details of participants’ ages, educational background, and employment status are presented in Table 1. All participants had normal or corrected-to-normal visual acuity; none reported any difficulty in making color discriminations. Stimuli were displayed on a 15-in. LCD computer display at a viewing distance of 45 cm. Participants performed a dual-feature working memory task (Bays, Wu, & Husain, 2011), which enabled us to quantify the precision of recall for visual features in two dimensions: color and orientation.[Table-anchor tbl1]

**Figure 1 fig1:**
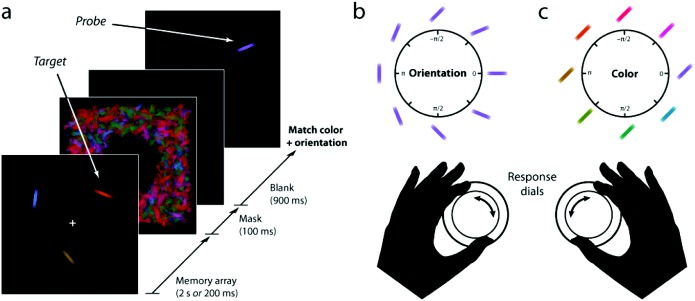
The dual-feature working memory task. (a) Participants were presented with an array of colored, oriented bars, followed by a pattern mask. After a blank retention interval, a *probe* appeared and subjects used two response dials to adjust its color and orientation to match the item at the corresponding location in the memory array (the *target*). (b & c) Turning each dial cycled the probe through a circular parameter space of possible colors or orientations. Some examples of orientations (b) and colors (c) are shown corresponding to different points in each response space.

Each trial began with presentation of a memory array (example in Figure 1a) consisting of randomly colored and oriented bars (0.64° × 3.6° of visual angle) uniformly distributed around an imaginary circle (8° diameter) centered in the display (minimum interitem separation: 6.9°). Half of the participants were shown each memory array for 2 s and the other half for 200 ms, with the two groups matched for age distribution (*p* = .94, two-sample Kolmogorov–Smirnov test). The memory array was followed by a pattern mask (100 ms) and then a blank retention interval (900 ms). The pattern mask was included to ensure iconic (sensory) memory did not contribute to performance. A single (*probe*) item was then presented at one randomly chosen location from the preceding memory array. Subjects were instructed to adjust the orientation and color of the probe item to match the features of the item that had been presented at the same location in the memory array (the *target*).[Fig-anchor fig1]

The color and orientation of each item in the memory array were independently chosen at random from two circular parameter spaces. The orientation parameter space corresponded to the range of angles 0°–180° (i.e., the full range of possible bar orientations). For color, the parameter space was defined by a circle in CIE L*a*b* coordinates with constant luminance (L* = 50), center at a* = b* = 20, and radius 60.

Participants adjusted the color and the orientation of the probe using two input dials (Powermate USB Multimedia controller, Griffin Technology, Nashville, TN). One dial controlled the probe’s color and the other dial its orientation. Turning the dial associated with orientation caused the probe to rotate through the range of possible angles (Figure 1b). Turning the dial associated with the color feature caused the probe to cycle through the space of possible colors (Figure 1c). The probe’s initial features were randomly assigned. Subjects could adjust the two dials in any order or simultaneously, and indicated adjustment was complete by depressing the center of either dial. Accuracy was stressed, and responses were not timed.

Two memory loads were tested: a *low-load* condition, in which one item only was presented in each trial, and a *high-load* condition, in which three items were presented per trial. In the *high-load* condition the item to be probed was randomly selected on each trial, so participants were required to memorize the color and the orientation of all three bars in order to perform well on the task. Each participant completed 25 trials in the *low-load* condition, and at least 125 trials (median 175) in the *high-load* condition. Fewer trials were required in the low-load condition because there was no possibility of misreporting a nontarget item, simplifying the data-intensive modeling component of the analysis (see below).

### Standard Tests of Memory Span

In addition to the main experiment, each participant also completed a set of well-established tests of STM span: the forward and backward digit span tasks, and the forward and backward Corsi block-tapping tasks for spatial span. In the digit span tasks, participants were read sequences of digits (e.g., 3, 7, 9, 4) and asked to repeat them back in the same order (forward digit span task) or in the reverse order (backward digit span task). Each test began with sequences of two digits. If participants performed successfully, they were asked to repeat longer sequences. A participant’s score (or “memory span”) was defined as the maximum length at which they could successfully recall at least one out of two sequences. In the Corsi spatial span task, the experimenter pointed in sequence to a set of cubes randomly distributed on a platform and participants are asked to reproduce the sequence either in the same order (forward Corsi task) or in the reverse order (backward Corsi task). Performance was scored in the same way as the digit span task. The sum of forward and backward scores was calculated to give a total score on each task, and the sum of these totals calculated to give a total test score.

### Analysis

The analysis of the dual-feature task closely followed that described by Bays, Wu, and Husain (2011) in their study of younger participants. As the spaces of possible values for both color and orientation were circular, feature values and participants’ responses were coded as angular measures (from −π to π radians). For each trial, the recall error in each dimension was calculated as the angular deviation between the reported feature value and the true feature value of the target item in the memory array. Recall precision was defined as the reciprocal of the circular standard deviation of error. We used the definition of standard deviation for circular data given by Fisher (1995), and subtracted from the precision estimate the value expected by chance (i.e., if the subject had responded at random on each trial).

To investigate the source of participants’ errors in the dual-feature task, we applied a probabilistic model developed by Bays et al. (2009), extending the previous model of Zhang and Luck (2008). In our model, error in reporting the value of a particular feature, for example, orientation, is assumed to arise from three possible sources (illustrated in Figure 4a): Gaussian variability in reporting the feature value belonging to the target; mistakenly reporting a feature value of one of the other (nontarget) items in the memory array; or simply responding at random. Note that in Zhang and Luck’s (2008) model, misreporting nontarget features—an index of misbinding—is not modeled.[Fig-anchor fig4]

Mathematically the model is described by the following equation:

p(θ^)=αϕκ(θ^ − θ)+β 1m∑imϕκ(θ^ − φi)+γ 12π 1

where θ is the true value of the target item, $\hat{\theta }$ the value reported by the subject, and $\phi_{\kappa }$ is the von Mises distribution (the circular analogue of the Gaussian) with mean zero and concentration parameter κ. The probability of reporting the correct target item is given by α. The probability of mistakenly reporting a nontarget item is given by β, and $\left\{\varphi_{1}^{},\;\varphi_{2}^{},\;...\;\varphi_{m}^{}\right\}$ are the feature values of the *m* nontarget items. The probability of responding randomly (i.e., from a uniform distribution) is given by γ=1−α−β.

Maximum likelihood estimates of the parameters α, β, γ and κ were obtained separately for each subject, feature dimension, and experimental condition using an expectation-maximization algorithm. The optimization procedure was repeated from a range of different initial parameter values to ensure that global maxima were obtained. Concentration κ was converted to the more familiar standard deviation, σ, according to the method of Fisher (1995). Analysis code is available online at http://www.sobell.ion.ucl.ac.uk/pbays/code/JV10/

We thus obtained separately for each participant and feature dimension (color and orientation) the probability of responding with the target value (*target* component), the probability of responding randomly (*uniform* component) and, in the *high-load* condition, the probability of responding with one of the two nontarget values (*nontarget* component). We also obtained from the model the standard deviation of the von Mises distribution, corresponding to the variability in memory for each feature dimension.

Data from each individual participant was analyzed separately to obtain the measures described above. Statistical outliers (defined as parameter values > 3 *SD* from mean, totaling < 2% of data points) were removed, then each parameter was tested for effects of age using linear regression. ANCOVA was used to make statistical comparisons between conditions (*low-load* or *high-load*) and feature dimensions (*color* or *orientation*), with age as a continuous covariate. As participants’ ages were approximately evenly distributed over the age range, for the purposes of presentation only we divided the sample into four age quartiles. Demographics of the different groups thus formed are presented in Table 1.

## Results

### Recall Errors and Precision

**Figure 2 fig2:**
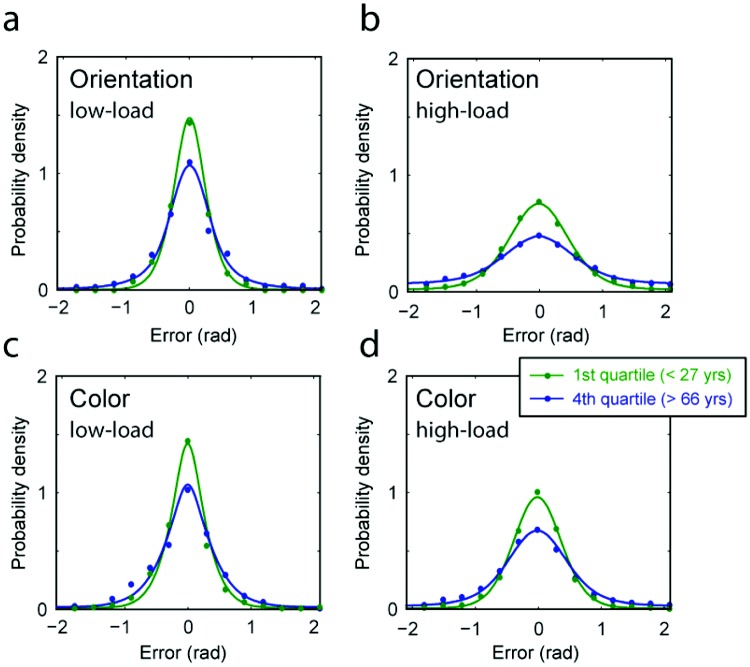
Distribution of errors for young and old participants. (a–b) Frequency of response as a function of the deviation between reported and target *orientations*, for participants in the youngest (green symbols) and oldest (blue symbols) age quartiles. Results are shown for [low-load, one memory item, (a); high-load, three memory items, (b)] conditions. Colored lines indicate the response probabilities predicted by a fitted probabilistic model of response generation. Note the increase in response variability (width of the distribution) for older participants. (c–d) Corresponding results for recall of *color*.

Adults of different ages were tested on their ability to reproduce from memory the color and orientation of a single probed item from an array of colored bars (see Figure 1). Figure 2 illustrates the distribution of errors made on this task by the youngest participants (lowest age quartile, < 27 years, green) and the oldest (highest age quartile, > 66 years, blue) for each feature dimension—orientation or color—and memory load (one or three items). In each case, older participants produced a broader distribution of errors indicating less accurate recall of the memory array.[Fig-anchor fig2]

**Figure 3 fig3:**
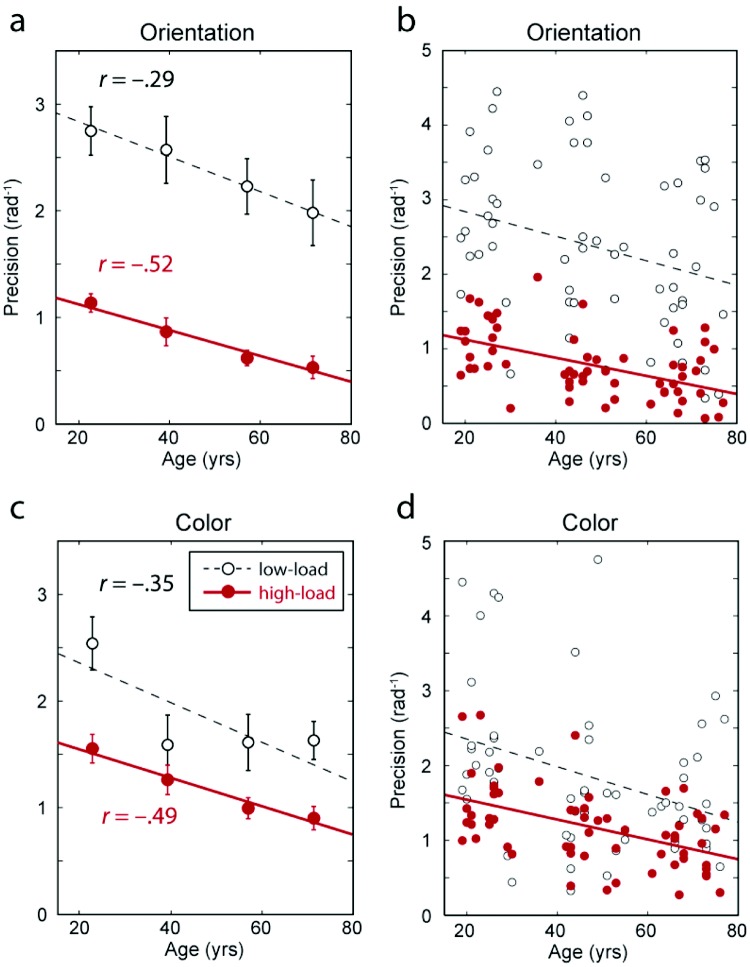
Age effects on recall precision. (a–b) Precision of *orientation* recall as a function of age, for low-load (black) and high-load (red) conditions. Symbols and errorbars in (a) indicate mean ± 1 *SE* for each age quartile. Individual subject results are plotted in (b). Precision is here defined as the reciprocal of the standard deviation of error in participants’ responses; zero indicates chance performance. Fitted regression lines are shown for the relationship between precision and age. (c–d) Corresponding results for *color* recall.

The fidelity of memory can be formally assessed with a measure of recall precision (see Figure 3), which evaluates the degree to which responses cluster around the correct feature value: A precision of zero indicates that responses are randomly distributed relative to the target. Consistent with previous studies involving only younger participants, for all age quartiles recall precision decreased significantly with increasing memory load (compare Figures 2a & 2c with Figures 2b & 2d; red vs. black symbols in Figure 3; main effect of *load*: *F*(1, 58) = 22.6, *p* < .001).[Fig-anchor fig3]

In the *high-load* condition, when participants had to maintain three items in memory simultaneously, recall precision declined significantly with increasing age (main effect of *age*: *F*(1, 58) = 17.7, *p* < .001). This effect of age was present both for orientation (*F*(1, 58) = 21.2, *p* < .001) and color judgments (*F*(1, 58) = 18.5, *p* < .001). A significant effect of age was also observed in the *low-load* condition, when only one item had to be maintained (main effect: *F*(1, 58) = 5.9, *p* = .017; for orientation: *F*(1, 58) = 34.8, *p* = .025; for color: *F*(1, 58) = 8.4, *p* = .006). The relationship between age and precision was significantly stronger in *high-load* than *low-load* conditions (*r* = −0.50 vs. −0.32; *t*(57) = 2.1, *p* = .04). There were no significant effects of feature (color vs. orientation) or age × feature interactions.

### Components of Error

**Figure 4 fig4:**
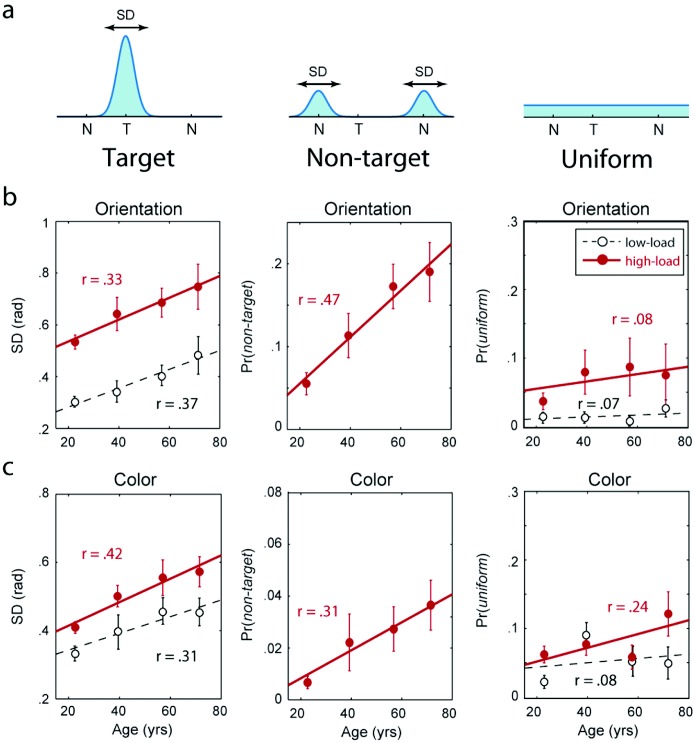
Components of error in the working memory task. (a) The distribution of responses in each feature dimension was decomposed into a mixture of three separate components: responses distributed with Gaussian variability around the correct target (T) feature value (left), responses distributed around the feature values of other, nontarget (NT) items in the memory array (center), and responses uniformly distributed throughout the response space (right). (b–c) Maximum likelihood estimates of parameters of the mixture model illustrated above, for *orientation* responses (b) and color responses (c). Fitted regression lines illustrate relationships between each model parameter and age. Gaussian variability in target responses (left) increases with age, as does the frequency of nontarget responses in multiitem arrays (center). The frequency of random responses (right) is not correlated with age. Error bars indicate ± 1 *SE*.

These results suggest that the fidelity with which individual features can be recalled declines significantly across the adult life span. This conclusion is based on the relationship between age and precision, a nonparametric statistic reflecting the fidelity of recall of the target features, independent of any particular model of the underlying response distribution. To investigate the *source* of this overall decline in precision, we fit the data with a probabilistic model that distinguishes three sources or components of error (illustrated in Figure 4a). These components are:

• Gaussian variability in recall centered on the correct target feature (left panels in Figure 4);
• Misreporting errors, in which participants report a feature belonging to one of the other (nontarget) items (center panels, Figure 4);
• Uniformly distributed errors unrelated to any of the items in the display (right panels, Figure 4).

The fitted parameters of the model are shown in Figures 4b and 4c, for orientation and color judgments, respectively. Left-hand panels plot the standard deviation of the Gaussian component of the fitted model, indicating the variability with which each feature was recalled. Consistent with the overall performance effects described above, recall variability increased significantly with age in the high-load condition (main effect of *age*, *F*(1, 58) = 6.3, *p* = .013; for orientation only: *F*(1, 58) = 4.41, *p* = .04; for color only: *F*(1, 58) = 12.3, *p* < .001). A significant effect of age on variability was also observed in the low-load condition (main effect, *F*(1, 58) = 10.9, *p* = .001; for orientation: *F*(1, 58) = 9.6, *p* = .003; for color: *F*(1, 58) = 5.3, *p* = .025). Gaussian variability increased significantly with increasing memory load (main effect of *load*: *F*(1, 58) = 7.8, *p* = .006).

Consistent with previous results in young adults, a significant proportion of responses in multiitem arrays (mean 23% for orientation, 12% for color) were *not* accounted for by Gaussian variability centered on the target feature. These responses were instead attributed in the model to either misreporting or uniformly distributed errors (Bays et al., 2009; Bays, Wu, & Husain, 2011; Zhang & Luck, 2008).

The center panels in Figures 4b and 4c show the frequency of misreporting errors as estimated by the model, for orientation and color judgments, respectively. These errors occur in *high-load* (i.e., multiitem) arrays when the participant incorrectly reports a feature belonging to one of the other, nontarget items (by definition such errors cannot occur in the *low-load* condition, when only one item had to be retained). Although these errors were rare for the youngest participants (mean 5% for orientation, 1% for color), the frequency of misreporting errors increased significantly with age (main effect of *age*: *F*(1, 58) = 30.3, *p* < .001; for orientation only, *F*(1, 58) = 16.8, *p* < .001; for color only, *F*(1, 58) = 6.3, *p* = .017). Although significant for both features, the increase in misreporting errors was greater for orientation (mean 19% for highest age quartile) than for color (mean 4%) as confirmed by a significant age × feature interaction, *F*(1, 58) = 9.6, *p* = .002.

Finally, we turn to uniformly distributed errors. Right-hand panels in Figure 4 show the frequency of these responses. Unlike misreporting errors, the frequency of uniform errors was not influenced by age, either for *low-load* (main effect of *age*: *F*(1, 58) = 0.1, *p* = .73) or *high-load* arrays (main effect of *age*: *F*(1, 58) = 0.5, *p* = .48). Uniform error frequency was also not significantly affected by load (main effect of *load*: *F*(1, 58) = 0.7, *p* = .40).

### Effect of Exposure Duration

Previous studies examining recall precision in young adults have found that limiting the duration of presentation of the memory array leads to a decrease in recall fidelity (Bays et al., 2009; Bays, Gorgoraptis, et al., 2011). With respect to components of the probabilistic model, brief masked exposures lead to an increase in both Gaussian variability and the frequency of uniformly distributed errors, consistent with incomplete encoding of array items into memory (Bays, Gorgoraptis, et al., 2011).

**Figure 5 fig5:**
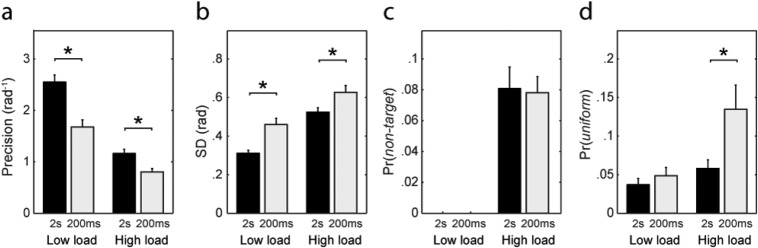
Effects of exposure duration on recall precision and error components. (a) Precision of recall for static (2 s; black bars) and briefly flashed (200 ms; light bars) memory arrays, in low- and high-load conditions. Asterisks indicate significant (*p* < .05) effects of exposure duration. Results shown are means over feature dimensions. (b–d) Effects of exposure duration on components of error as specified by the mixture model: (b) Gaussian variability (*SD*), (c) frequency of misbinding (nontarget) errors, (d) uniformly distributed errors.

In the present experiment, presentation duration was manipulated as a between-subjects factor. Figure 5a plots precision as a function of exposure duration for low-load and high-load displays (average across feature dimensions). Consistent with previous studies, presenting the array briefly (200 ms) resulted in a significant decrease in precision for both array sizes, *t*(58) > 3.3, *p* < .002, compared with a longer presentation time of 2 s. Figures 5b–5d show the effect of exposure duration on each of the parameters of the mixture model. Shorter exposures resulted in significantly increased Gaussian variability at both high and low loads, *t*(58) > 2.3, *p* < .022. Decreased exposure time also significantly increased uniform responses when multiple items were presented (high-load: *t*(58) = 2.3, *p* = .025), but not for displays of one item (low-load: *t*(58) = 0.87, *p* = .39). There was no significant effect on frequency of misreporting errors, *t*(58) = 0.16, *p* = .87.[Fig-anchor fig5]

We observed no significant effect of exposure duration on correlations between age and precision, or between age and any of the parameters of the probabilistic model. The significant effects of age on precision, Gaussian variability, and misreporting frequency observed in the main analysis were reproduced when both long (2 s) and short (200 ms) exposure groups were analyzed separately (*p* < .05).

In summary, although decreasing exposure duration had the expected detrimental effect on recall performance for subjects regardless of age, the decline in recall fidelity with age was a consistent finding at both long and short exposures.

### Nonparametric Validation of Misreporting Errors

The parameter estimates obtained from fitting the probabilistic model indicate that older participants were more likely to incorrectly report features of nontarget items (Figure 4, center panels). To demonstrate that this result is not dependent on specific details of the model, and provide a more direct test of the presence of misreporting errors, we examined the deviations between subjects’ responses and the feature values of nontarget items.

For data consisting only of responses to the target, or random guesses, or a mixture of the two, we would expect the deviation of responses from nontarget features to be randomly distributed. However, the magnitude of deviations from nontarget features observed in our data (1.78 ± 0.01 rad, r.m.s.) was significantly lower than expected by chance, *t*(59) = 4.9, *p* < .001, confirming that nontarget features contributed significantly to our subjects’ responses overall.

If frequency of nontarget errors increases with age, as the model parameters indicate, we would expect to see a corresponding decrease in the deviation of responses from nontarget features. The magnitude of deviations from nontarget features indeed declined significantly with age (*r* = −0.45, *p* = .001), providing an additional nonparametric validation of the age-related increase in misreporting errors indicated by model fitting.

### Joint Distribution of Errors

Because participants reported both color and orientation on each trial, we can examine the extent to which errors in the two different features are correlated. Based on performance of younger participants on the dual-feature task, Bays, Wu, and Husain (2011) demonstrated substantial independence between features in both the magnitude and source of errors, a finding with important implications for the structure of working memory representations (see also Fougnie & Alvarez, 2011). The present study presents the opportunity to investigate whether these results are replicated in a larger sample covering a broader range of ages.

**Figure 6 fig6:**
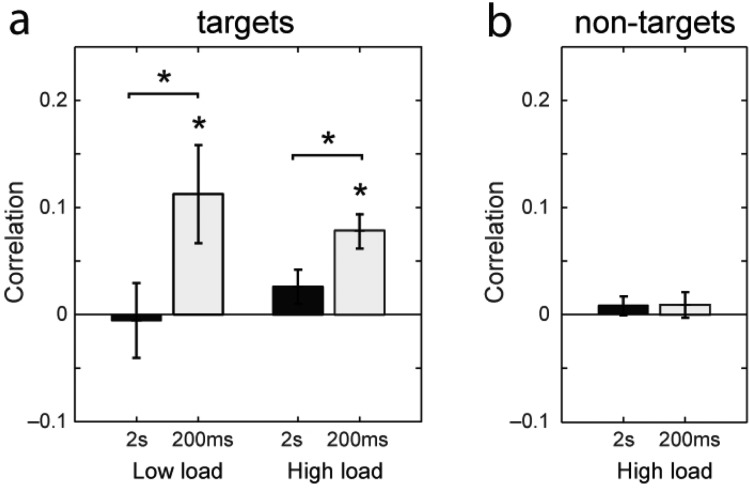
Correlation of errors in orientation and color judgments. (a) Correlation between magnitude of error in color and orientation judgments for static (2 s; black bars) and briefly flashed (200 ms; light bars) memory arrays, in low- and high-load conditions. Asterisks indicate significant (*p* < .05) correlations and effects of exposure duration. (b) Correlation between magnitude of deviation of responses from *nontarget* colors and orientations in the high-load condition, for static and briefly flashed memory arrays. No significant correlations were observed, indicating that misreporting of nontarget features occurred independently in each feature dimension.

Figure 6a displays the correlation in error magnitude between color and orientation judgments for different array sizes and exposure durations. Consistent with previous studies, with prolonged exposure (2 s) no significant correlations were observed in *low-* or *high-load* conditions (black bars; *t*(59) < 1.6, *p* > .12). This means that the size of the error in recalling one feature of an item was fully independent of the size of the error in recalling the other feature of the item, on the same trial.[Fig-anchor fig6]

Bays, Wu, and Husain (2011) hypothesized that very brief presentation times could produce correlated errors, if there was insufficient time to encode all items in the memory array. Consistent with this, in the brief (200 ms) exposure condition we observed a small (*r* < .1) but significant correlation in error magnitude between features (light bars; *t*(59) > 2.4, *p* < .021; effect of duration: *t*(59) > 2.0, *p* < .046).

In their study of young adults, Bays et al. observed that misreporting errors also occurred independently in each feature dimension, that is, the fact that a participant reported a nontarget’s color did not make it any more likely that they would incorrectly report the same nontarget’s orientation (or vice versa). If misreporting errors were correlated across feature dimensions in the present study, it would be observed as a correlation in the magnitude of the response deviation from nontarget feature values (Bays, Gorgoraptis, et al., 2011). This correlation did not differ significantly from zero for long or short exposures (Figure 6b; *t*(59) < 1.1, *p* > .32), and was significantly lower than expected if nontarget errors in each dimension occurred together (*t*(59) > 3.2, *p* < .003; based on marginal frequencies obtained from model parameters). These results indicate that—like in the previous study—misreporting errors occurred independently for color and orientation judgments.

### Memory Span Tests

Participants completed two standard tests of working memory span: digit and Corsi spatial span. Both tasks were executed forward and backward. The results for each age quartile are presented in Table 1. Total score on the memory span tasks was correlated both with age (*r* = −0.34, *p* = .009) and with the number of years participants had spent in full-time education (*r* = .49, *p* = .001; see Table 2). However the correlation with age became nonsignificant once the influence of education was removed (partial correlation: *r* = −0.16, *p* = .24). By contrast, low-load precision in our experimental task was not correlated with years of education (*r* < .24, *p* > .07), and although high-load precision was correlated with education (for orientation: *r* = .39, *p* = .002; for color: *r* = .33, *p* = .01) these correlations became nonsignificant once the influence of age was removed (partial correlation: *r* < .21, *p* > .11).[Table-anchor tbl2]

Significant correlations were found between total score on the memory span tasks and recall precision in both the high-load and the low-load conditions of our experimental task, for both orientation (for the low-load condition: *r* = .42, *p* < .001; for the high-load condition: *r* = .67, *p* < .001) and color (for the low-load condition: *r* = .44, *p* < .001; for the high-load condition: *r* = .58, *p* < .001; see Table 3). Each individual component of the Corsi spatial task (forward and backward) was also correlated with high-load precision (*p* < .001) and low-load precision (*p* < .005). The forward and backward components of the digit span task were correlated with high-load precision (*p* < .002), but only the backward component was correlated with low-load precision (*p* = .02).[Table-anchor tbl3]

Correlations were stronger between memory span scores and high-load precision than between memory span scores and low-load precision. The component that correlated most strongly with high-load precision was the reverse Corsi task (for orientation: *r* = .60, *p* < .001; for color: *r* = .56, *p* < .001). Forward spans were more weakly correlated with precision than backward spans, and the forward digit span task was least strongly correlated (for high-load precision, orientation: *r* = .48, *p* < .001; for color: *r* = .40, *p* = .002).

It is important to test whether the significant correlations reported above between memory span and working memory precision are mediated by age. Total score on memory span tasks remained correlated with both high-load precision and low-load precision when the influence of age was removed (partial correlation: *p* < .001). Both forward and backward spatial span scores were correlated with high-load and low-load precision (forward: *p* < .05; backward: *p* < .005) when age was held constant, whereas for the digit span task, only the correlations with high-load precision remained significant (forward: *p* < .009; backward: *p* < .005).

## Discussion

Memory is one of the cognitive functions most affected by normal aging (Craik et al., 2010; D’Esposito & Gazzaley, 2011; Iachini et al., 2009; Light, 1991; Reuter-Lorenz & Sylvester, 2005; Salthouse & Babcock, 1991). Previous studies have shown that older adults’ performance in a variety of working memory tasks is poorer than younger adults’ (Bopp & Verhaeghen, 2009; Brockmole et al., 2008; Cowan et al., 2006; Mitchell et al., 2000; Parra, Abrahams, Logie, et al., 2009; Sander et al., 2011a). These studies have mostly attributed the deficit to a decrease in the *number* of objects that can be held in memory. Here we have isolated a different component of age-related memory decline: a decrease in the precision, or resolution, with which individual visual features can be recalled from visual working memory.

This deficit was observed as an increase in the variability with which older participants reproduced from memory both the color and orientation of visual objects. A significant increase in variability with age was observed when just a single object was held in memory. However, correlations with age were stronger when multiple items were maintained, suggesting that VWM deficits in older individuals may become more prominent under conditions of high memory load.

Significant correlations were observed between working memory precision and established tests of STM span (digit span and Corsi tasks). It is notable that precision was more closely correlated with backward than forward span tasks. The backward tasks are commonly considered to more directly tap the ability to manipulate maintained information (Cornoldi & Mammarella, 2008; Groeger, Field, & Hammond, 1999; Reynolds, 1997) although this distinction between backward and forward tasks is debated (Rosen & Engle, 1997). The stronger correlation with the Corsi spatial task over digit span is consistent with the visuospatial nature of the precision task.

In line with previous results (Dobbs & Rule, 1989), performance on the standard tests was found to be more strongly and directly correlated with participants’ education than with their age. By contrast, working memory precision displayed a strong relationship with age but was not directly influenced by number of years in education. In this respect, the precision measure potentially provides a more direct assessment of age-related cognitive decline than these commonly used tests of WM ability.

As the adjustment of the probe item was not timed, limits on motor performance were not expected to influence the precision of reproduction on this task. Furthermore, the possibility that deterioration in motor accuracy is responsible for the present results is effectively negated by the observation that effects of age were strongest in the higher memory load condition. Because the response stage of *low-* and *high-load* conditions was identical, any increase in variability due to motor impairment would affect both conditions equally. Furthermore, because the baseline (young adult) precision was higher in the one-item condition, effects of motor impairment would be most apparent in this condition, contrary to the present results.

A recent study of school-age children has observed improvements in VWM precision with age during middle childhood and early adolescence (Burnett Heyes et al., 2012). In combination with the present results, these findings suggest that changes in WM precision may closely track cognitive development and decline across the life span.

### Binding Errors in Older Adults

Previous studies have assessed the effect of aging on visual working memory using binary (correct or incorrect) measures of recall, in particular the change detection method in which participants judge sequential pairs of images to be the same or different (Luck & Vogel, 1997; Pashler, 1988; Vogel et al., 2001). These studies have consistently found a deterioration of performance in older participants. However, the underlying basis of this deficit is controversial, particularly with respect to the role of *binding*, or associating individual features with objects (Treisman, 1998; Wheeler & Treisman, 2002).

Cowan, Naveh-Benjamin, Kilb, and Saults (2006) presented pairs of arrays of colored squares that could differ either in the addition of a new color (feature change) or a change in which color belongs with which object (conjunction change). A performance cost that was specific to older participants was observed in the conjunction change condition, suggesting that a deterioration in the ability to maintain binding information may be a separable component of age-related decline in VWM.

This finding accords with a number of studies that have shown age-related deficits in maintaining associations in *long-term* memory (Chalfonte & Johnson, 1996; Naveh-Benjamin, 2000; Naveh-Benjamin, Guez, Kilb, & Reedy, 2004). However, other studies using similar methods have failed to corroborate a binding deficit in VWM in older individuals (Brockmole et al., 2008; Parra, Abrahams, Logie, et al., 2009). Although these studies have observed poorer performance for older participants in conjunction conditions, they have observed equivalent deficits in conditions thought not to require feature binding, suggesting that VWM binding may not be a specific target of age-related decline.

Assessing binding deficits in these previous studies has required a comparison of frequency of errors in at least two separate conditions. To take a typical example, Parra, Abrahams, Logie, et al., (2009) compared change detection accuracy under conditions where changes were to a single color versus changes to the conjunction of color-pairs. Performance differed between these conditions in both young and old participants, and *both* conditions were impaired in older participants. Testing for an age-specific binding impairment therefore depended on assessing whether the *difference* in performance between feature and conjunction conditions was greater in old than young participants, and hence a subtraction of error frequencies (or other performance measures) across conditions. The meaningfulness of this operation is unclear, and difficult to assess in the absence of a full mathematical treatment of how errors are generated on the task.

By contrast, in the present study a measure of the frequency of misbinding was obtained from responses on a single task in which observers directly reported remembered features of a probed item. In the *high-load* condition, participants were required to report the color and orientation of one item from a three-item memory array. The item to report was indicated by a location cue (the probe). So, if errors occurred in binding features and locations together into objects, we would expect to see trials in which participants incorrectly reported a color or orientation belonging to an item at a different location to the probe. A previously developed probabilistic model (Bays et al., 2009) provides the frequency of these misreporting errors as one of its parameters. The results showed that, although rare in younger participants at these memory loads, misreporting errors indeed made up a substantial minority of responses for older individuals.

The modeling approach allowed us to place a numerical estimate on the frequency with which nontarget features are reported, by specifying a circular Gaussian distribution of error around reported feature values. We also conducted a separate analysis that did not assume any particular error distribution, but was instead based simply on the average deviation of responses from nontarget features. This nonparametric analysis also confirmed a significant increase in nontarget influence with age.

Previous studies based on change detection have differed in whether changes in the conjunction condition affected the pairing of features with locations (e.g., Cowan et al., 2006; Olson et al., 2004), the pairing of features with other nonspatial features (e.g., Parra, Abrahams, Logie, et al., (2009); Parra, Abrahams, Logie, & Della Sala, 2010), or both (e.g., Brockmole et al., 2008). In the present study, our misreporting estimate reflected the ability of subjects to correctly identify which visual features corresponded to a particular probed location. However, as with previous studies, we should be wary of concluding that performance reflects only the recall fidelity of feature-location binding. For example, the color corresponding to a location could be correctly identified by first recalling the orientation presented at that location, and then the color paired with that orientation (although note that the absence of a significant correlation of misreporting errors between features may constrain the role for this kind of inference). Future work should address the extent to which location plays a special role in binding visual features, and seek to distinguish failures of feature–location binding from failures of feature–feature binding in healthy and aging populations.

A recent study (Campbell, Hasher, & Thomas, 2010) using a paired-associates memory task found increased priming from task-irrelevant distractors in older participants compared with young adults. This result is consistent with behavioral results suggesting older individuals may be impaired in inhibiting irrelevant inputs to working memory (Cashdollar et al., 2012; Hasher, Zacks, & May, 1999), and EEG studies pointing to an age-related decline in top-down control over memory allocation (Jost, Bryck, Vogel, & Mayr, 2011; Sander, Werkle-Bergner, & Lindenberger, 2011b, 2012). A consequence of such deficits is that irrelevant material may enter working memory, potentially accompanied with binding information relating it to simultaneously presented task-relevant inputs. Contrastingly, in the present study all features and objects presented to subjects were of equal importance to the task and required maintenance in memory. So, although impairments in filtering out task-irrelevant information may contribute to cognitive decline in older individuals, they are unlikely to have had a significant impact on the present results, which we suggest reflect changes with age in the maximum fidelity with which information can be encoded into working memory.

Emrich and Ferber (2012), using the same modeling approach as the present study, examined the effects of interitem separation on misreporting errors. When memory arrays were flashed briefly (100 ms or 200 ms), presenting targets in closer proximity increased the frequency of nontarget reports. This effect was reduced when competing items were distributed between two sequentially presented arrays, decreasing competition at the encoding stage. These results suggest that nontarget error frequency may be inflated in brief exposures of crowded displays as a result of incomplete or errorful encoding of the display into working memory. Another study that parametrically varied exposure duration in a reproduction task (Bays, Gorgoraptis, et al., 2011) also found evidence for an increase in misreporting responses with very brief (≤ 50 ms) masked exposures. However, the main factor determining nontarget error frequency was the number of items in the array: Increasing memory load led to more misreporting errors irrespective of exposure duration (see also Bays et al., 2009).

In the present study, we used relatively widely separated items (7° min. separation, comparable with the “low-competition” condition in Emrich & Ferber, 2012). Comparison of brief (200 ms) and prolonged (2 s) array presentations showed no effect of exposure duration on misreporting frequency. The fact that these errors are common even when observers have the opportunity to study the array for a prolonged period suggests that they arise principally from limits on how much information can be stored or maintained in VWM. Although competition at the encoding stage may contribute to misreporting errors in briefly presented crowded arrays, it does not appear to have been a significant factor in the present study.

Although misreporting errors were unaffected, reducing the time available to encode the memory arrays nonetheless decreased the accuracy with which participants reported visual features. In terms of the probabilistic model, this performance cost reflected an increase in the variability of responses around the correct target value (Figure 5b) and, in *high-load* arrays, an increase in the uniform parameter corresponding to random responses (Figure 5d). These findings again corroborate those previously obtained for university-age subjects by Bays, Gorgoraptis, et al. (2011), who argued that errors in recall of briefly flashed arrays reflect limits on the rate at which information can be encoded into memory, in addition to limits on memory capacity.

The present results may have important implications in relation to recent studies that have proposed misbinding as a marker for Alzheimer’s disease (Parra, Abrahams, Fabi, et al., 2009; Parra, Abrahams, Logie, & Della Sala, 2010; Parra, Abrahams, Logie, Méndez, et al., 2010). Those studies, based on the change detection paradigm, have observed substantial deficits in conjunction conditions in Alzheimer patients and asymptomatic carriers of a mutation associated with the familial form of the disease. Although the binding deficit may indeed be stronger in these individuals than in age-matched controls, the observation here of substantial misbinding in older participants chosen from the general population urges caution in interpreting the mere presence of misbinding as diagnostic of Alzheimer’s disease in older individuals. It nevertheless remains possible that there are quantitative differences in binding impairments between healthy, elderly people and those with Alzheimer’s.

### Working Memory Resources

The present results confirm and expand on a number of observations made previously in younger (university-age) participants (Alvarez & Cavanagh, 2004; Bays et al., 2009; Bays & Husain, 2008; Gorgoraptis et al., 2011; Lakha & Wright, 2004; Palmer, 1990; Wilken & Ma, 2004). First, we have demonstrated here, for a wide range of ages, that the fidelity with which visual features are recalled declines with increasing memory load (Figures 2 and 3), and further that a key component of this loss of fidelity is an increase in the variability (or *SD*) of the distribution of responses centered on the target feature value (Figure 4, left column).

As noted in previous studies of young adults, this decline of fidelity with load is difficult to reconcile with the influential model of VWM that considers memory capacity to reflect a fixed number of independent memory “slots,” each storing a unique visual object (Cowan, 2001; Luck & Vogel, 1997; Pashler, 1988). Competing models instead make reference to a working memory *resource* that is *distributed* between elements of a visual scene, with the result that when more items are stored, each is recalled with less precision (Bays & Husain, 2008; van den Berg, Shin, Chou, George, & Ma, 2012).

The distribution of errors on recall tasks has become an important topic for distinguishing competing models of VWM. In particular, the observation that the distribution of errors around target feature values deviates from Gaussian (or its circular equivalent) has been interpreted as evidence that a proportion of objects are not stored (Anderson, Vogel, & Awh, 2011; Zhang & Luck, 2008). According to these investigators, there is an upper limit on the number of items that can be held in VWM, either in addition to resource limitations (Anderson et al., 2011) or as the result of sharing out a quantized resource between objects (Zhang & Luck, 2008). For memory arrays that exceed this upper limit, there is a fixed probability on any trial that a probed object will not have gained access to memory, forcing the observer to guess at random as to the object’s features. By fitting response data from university-age subjects with a mixture of Gaussian and uniform distributions, these investigators obtained frequencies of “random” responses consistent with an upper limit at two or three objects.

The assumption that deviations of error distributions from Gaussian should be interpreted as an upper limit on objects stored has been questioned on a number of grounds (Bays et al., 2009; Bays, Gorgoraptis, et al., 2011; Bays, Wu, & Husain, 2011; Fougnie & Alvarez, 2011; van den Berg et al., 2012). Nonetheless, it is important to consider whether the effects of age on performance observed in our study could be explained by a failure to store one or more of the array objects. This seems unlikely in the low-load condition, where only one item had to be stored in memory, yet a significant decline in precision with age was observed. But in the high-load condition, subjects had to hold three objects in memory, equivalent to the typical upper limit claimed for university-age subjects by Zhang and Luck (2008), and hence potentially exceeding the capacity of older subjects.

To test this possibility, the probabilistic model used to fit the data (Bays et al., 2009) included a uniform component. We found no significant effect of age on the frequency estimated for uniform errors (Figure 4, right-hand panels), indicating that random guessing was not responsible for the age-related decline in memory performance observed here. Instead, poor recall in older participants resulted from a combination of increased variability in responses centered on the target value (consistent with a decline in memory resolution) and an increase in incorrect reports of nontarget features (consistent with an increase in binding failures).

Some previous studies based on change detection tasks (e.g., Cowan et al., 2006) have concluded that aging decreases the number of items that can be maintained in memory. However, the change detection methodology cannot distinguish between errors caused by unstored items and errors caused by variability in representation of stored items, of the kind demonstrated here. In young adults, a resource model based on Gaussian variability in memory representations successfully reproduces the error frequencies typically observed on change detection tasks, without recourse to an upper limit on items stored (Bays & Husain, 2008; see also Wilken & Ma, 2004; van den Berg et al., 2012). Similarly, it is possible that the deleterious effects of age on precision and binding observed here may be sufficient to account for age-related impairments on change detection tasks, without a change in how many items are stored. However, we cannot rule out the possibility that young and old observers differ in the frequency of random responding at set sizes larger than those tested here.

A recent study by Noack, Lövdén, and Lindenberger (2012) compared performance of younger and older participants on a working memory discrimination task (Bays & Husain, 2008). Unlike the present study, which examined recall of two nonspatial features (color and orientation), participants in the study of Noack et al. (2012) were tested on memory for object locations. A model-fitting procedure, similar to Zhang and Luck (2008), was used to partition responses into Gaussian-distributed errors and random guesses. Consistent with the present results, this study observed a significant increase in Gaussian variability with age. In addition, the older group was found to have a larger random component of responses, which Noack et al. (2012) interpreted as a decrease in the number of items stored. However, unlike the present study, their analysis did not consider the possibility that responses could have arisen from incorrectly responding based on the location of a nontarget item (i.e., misreporting errors). Based on the present results we would predict an increase in misbinding color and location in the older group that could account for some or all of the increase in the “guessing” component in this study.

Comparing model parameters for recall of color and orientation in the present study revealed a recall advantage for color, both in terms of the variability of the Gaussian-distributed component of error and the frequency of misreporting errors. This may represent a stable difference in the fidelity of internal representation of the two different feature dimensions. Alternatively, it is possible given the demanding nature of the task that subjects may have chosen to focus more resources on encoding and maintaining color than orientation information. Previous studies using the change detection methodology have found little or no performance cost associated with increasing the number of feature dimensions in memory (in contrast to increasing the number of features within a dimension), suggesting that different dimensions do not compete for the same VWM resources (Luck & Vogel, 1997; Wheeler & Treisman, 2002). However, one recent study using a continuous reproduction design has observed small effects of increasing feature dimensions on recall precision that may indicate a very limited ability to trade storage of one dimension off against another (Fougnie, Asplund, & Marois, 2010).

Alternatively, the performance advantages for color over orientation observed here may have arisen independently of changes in storage fidelity, for example, as a result of enhanced attention to color selection during the response stage of the task. Importantly, despite differences in recall fidelity between features, significant age-related declines in precision and binding were separately observed in both color and orientation responses.

A comparison can be drawn between the effects of age on memory performance observed here and the effects of memory load. Increasing the *number of items* in memory increases both the variability with which each item is stored and the frequency of misreporting errors (Bays et al., 2009; Bays, Gorgoraptis, et al., 2011; Bays, Wu, & Husain, 2011; Gorgoraptis et al., 2011). In the context of a resource model, these effects are attributed to a reduction in the WM resources available to maintain each item, resulting in a loss of fidelity in the storage of both individual features and the information that binds features into objects. Here we have observed that *increasing age* in the adult population similarly increases both variability in recall and misbinding frequency, suggesting that normal aging may be associated with a decline in working memory resources.

Both working memory variability and misbinding increase continuously with memory load in a manner consistent with the concept of a resource shared between visual objects. Also supporting the resource analogy, it is possible to “trade-off” memory precision between objects, so that behaviorally important items can be stored with enhanced precision at the cost of increasing variability for other items (Bays & Husain, 2008; Bays, Gorgoraptis, et al., 2011; Gorgoraptis et al., 2011). Although the mechanisms of storage and retrieval may differ between features and bindings, we hypothesize that the common element is that greater resource results in greater distinguishabililty of a representation from noise. In retrieval of an individual feature such as orientation, this means less *variability* in the recalled feature value; in retrieval of a binding between features (e.g., which of the orientations in memory corresponds to a particular location), this means a reduced probability of incorrectly retrieving a binding to the wrong feature.

The neural counterparts of working memory resources are yet to be identified. The age-related decline in resources observed here could reflect a decline in the number of neurons underlying working memory, or their responsiveness, or alterations in signaling across distributed neural networks. One recent study reported that prefrontal neural firing rates during visual working memory maintenance are reduced in aged monkeys, compared with their younger counterparts (Wang et al., 2011). Such decreases in the gain of activity in neural populations could reduce the ratio of signal to noise across a range of computations, consistent with the properties of a working memory resource outlined above.

In the study of Wang et al., 2011, the decline in activity could be reversed by iontophoretic application of guanfacine, a noradrenergic agonist. In humans, there is likely to be an increasing use of interventions—both drug and nonpharmacological—to improve working memory function in the elderly. Paradigms such as the one used here, based on a continuous, analogue response measure, might provide sensitive means by which to track the efficacy of such interventions.

## Conclusions

In this study, we examined the precision with which adult participants covering a wide range of ages were able to reproduce features of simple visual objects from memory. Increasing age was associated with a decrease in the resolution with which individual features were recalled, particularly under conditions of greater memory load. Aging was also linked to a significant increase in the frequency of errors related to *misbinding*, that is, errors in associating features with objects. These findings are consistent with a systematic decline in the working memory resources available to store visual information in older individuals.

## Figures and Tables

**Table 1 tbl1:** Participant Demographics and Mean Scores (SD in Parentheses) on Memory Span Tasks: Forward (F), Backward (B)

Quartile	1	2	3	4
Age (yrs)				
Range	19–26	27–46	47–66	67–77
*M* (*SD*)	22.7 (2.7)	39.3 (7.3)	57.1 (7.4)	71.5 (3.3)
Gender	10 F 5 M	5 F 10 M	11 F 4 M	9 F 6 M
Education (yrs)	15.5 (1.2)	14.5 (2.0)	15.0 (2.9)	12.4 (1.5)
% in education	80	7	0	0
% in employment	20	73	27	0
% retired/unemployed	0	20	73	100
Digit span				
F	11.1 (2.9)	9.9 (2.9)	10.5 (2.6)	8.9 (2.6)
B	8.0 (2.5)	6.3 (2.4)	7.6 (2.7)	5.9 (1.4)
Total	19.1 (5.2)	16.2 (4.9)	18.1 (5.1)	14.8 (3.5)
Corsi task				
F	8.3 (1.9)	7.3 (1.7)	7.3 (1.4)	7.0 (1.6)
B	7.7 (1.5)	6.7 (2.1)	6.5 (1.5)	6.1 (1.1)
Total	15.9 (1.5)	14.0 (3.3)	13.8 (1.9)	13.1 (2.3)
Total score	35.0 (6.5)	30.2 (6.8)	31.9 (6.1)	27.9 (4.9)

**Table 2 tbl2:** Correlations and Partial Correlations of Memory Task Performance With Age and Education

	Total score on memory span tasks	Precision in low-load condition	Precision in high-load condition
Correlation with age	−0.34	−0.40	−0.57
Correlation with education	0.49	0.23	0.40
Partial correlation with age (education held constant)	−0.16	−0.34	−0.48
Partial correlation with education (age held constant)	0.41	0.07	0.20

**Table 3 tbl3:** Pairwise Correlations for Memory Span Scores and Recall Precision. The Right (Upper) Triangle Shows Pairwise Correlations, the Left (Lower) Triangle Shows Partial Correlations With Age Held Constant

	Digit span total	Digit span forward	Digit span backward	Corsi span total	Corsi span forward	Corsi span backward	Precision low-load orientation	Precision low-load color	Precision high-load orientation	Precision high-load color
Digit span total		0.95	0.93	0.37	0.26	0.37	0.28	0.29	0.52	0.43
Digit span forward	0.94		0.75	0.35	0.25	0.35	0.23	0.26	0.48	0.40
Digit span backward	0.92	0.74		0.34	0.24	0.34	0.30	0.29	0.51	0.42
Corsi span total	0.31	0.30	0.29		0.85	0.84	0.49	0.49	0.64	0.58
Corsi span forward	0.20	0.19	0.18	0.84		0.44	0.41	0.37	0.49	0.42
Corsi span backward	0.32	0.30	0.29	0.82	0.38		0.42	0.47	0.60	0.56
Precision low-load orientation	0.22	0.18	0.24	0.43	0.35	0.36		0.32	0.63	0.40
Precision low-load color	0.23	0.20	0.23	0.42	0.29	0.41	0.24		0.52	0.72
Precision high-load orientation	0.48	0.44	0.47	0.57	0.41	0.54	0.58	0.42		0.66
Precision high-load color	0.38	0.34	0.36	0.49	0.33	0.49	0.31	0.68	0.54
